# Expression and correlation of the Pi3k/Akt pathway and VEGF in oral submucous fibrosis

**DOI:** 10.1111/cpr.13491

**Published:** 2023-05-08

**Authors:** Yanan Lin, Yueying Jiang, Haiyu Xian, Xiaoxiao Cai, Tao Wang

**Affiliations:** ^1^ Hainan General Hospital Haikou Hainan China; ^2^ The Affiliated Hainan Hospital of Hainan Medical University Haikou Hainan China; ^3^ State Key Laboratory of Oral Diseases, National Clinical Research Centre for Oral Diseases, West China Hospital of Stomatology Sichuan University Chengdu Sichuan China

## Abstract

Oral submucous fibrosis (OSF) has a high incidence in Asia countries, but its underlying molecular mechanism was not exploited completely. In this research, we investigated the expression of the phosphatidyl inositol 3‐kinase (Pi3k)/protein kinase B (Akt) pathway and vascular endothelial growth factor (VEGF) in oral submucosal fibrosis, explore the correlation between the Pi3k/Akt pathway and VEGF, and identify the mechanisms involved in OSF. The pathological changes and fibrosis stages of OSF tissues (*n* = 30, 10 each of early, moderate and advanced OSF) were determined using Haematoxylin–eosin staining (HE) and Masson staining, respectively. Collagen type I (Col‐I), Pi3k, Akt, VEGF, TGF‐β and p‐Akt expression was detected using immunohistochemistry, qPCR and WB. The correlation between Pi3k, Akt and VEGF was analysed. Col‐I expression increased as OSF progressed. However, their expression was downregulated in normal and moderate to advanced OSF tissues. VEGF expression positively correlated with Pi3k and Akt expression. VEGF expression correlated positively and negatively with the Pi3k inhibitor, LY294002 below and above a concentration of 10 μM, respectively. VEGF expression correlated positively with the Pi3k/Ak activator, IGF‐1. Due to the synergistic effect between Pi3k/Akt pathway and VEGF on OSF lesions and fibrosis process, targeted Pi3k/Akt pathway regulation can induce VEGF expression and improve ischemia, ultimately treating OSF.

## Graphical abstract

With the process of oral submucous fibrosis, the expression level of Collagen increased with rising inflammatory response. In vitro experiments also demonstrate that IGF‐1, the activator of Pi3k/Akt pathway promoted proliferation and migration of huvecs while LY294004, the inhibitor, exhibited the opposite function.

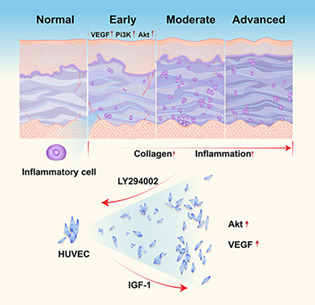

## 1 INTRODUCTION

The pathological manifestations of oral submucous fibrosis (OSF) include epithelial atrophy, inflammatory cell infiltration, collagen deposition and blood vessel loss, which eventually lead to structural and functional damage of the oral mucosa.^1^ Severe OSF limits mouth opening, causes dysphagia and is a cancer precursor transforming into a malignant tumour in 7%–30% of all cases.^2^, ^3^ VEGF, important in vasculogenesis and angiogenesis, acts an essential role in organism repair and fibrosis. VEGF regulates the function of the extracellular matrix through endothelial cell proliferation and migration blood vessel formation, and matrix metalloproteinase expression.^4^, ^5^ In addition, the expression of VEGF could be significantly increased through the up‐regulation of Pi3k/AKT signalling, a significant pathway in numerous cellular functions, like cell proliferation, survival and apoptosis.^6^, ^7^ According to previous researches, Pi3k/AKT pathway can promote VEGF expression and secretion, and play a role in angiogenesis.^8^ It effects the expression level of angiogenic factors including nitric oxide and angiopoietins. After the cells were disposed with inhibitors targeting the Pi3k/AKT pathway, VEGF secretion level showed an obvious decline. In this research, we explored the relationship between the Pi3k/Akt pathway and VEGF using immunohistochemistry, quantitative polymerase chain reaction (qPCR), and western blot (WB). We also aimed to identify the effects of an inhibitor and an activator on cell biological behaviours and the underlying mechanisms in the process of pathogenesis in OSF.

## 2 MATERIALS AND METHODS

### 2.1 Study participants

Between September 2019 and September 2020, 30 cases of buccal OSF (*n* = 10 each of early, moderately advanced, and advanced OSF) were clinically and pathologically diagnosed in the outpatient Department of Stomatology at Hainan General Hospital, Haikou, Hainan Province, China. After clinical diagnosis, the patients underwent pathological biopsy, haematoxylin and eosin (HE) staining and immunochemistry experiments were conducted on the obtained tissue, and staged according to Lindborg's OSF pathological staging criteria. Each diagnosis was made by two pathologists. The criteria for inclusion of participants were buccal OSF patients with a history of betel nut chewing, who did not have any other oral mucosal diseases and systemic disease, or did not undergo special treatment. Normal tissue samples (*n* = 10) were also obtained and served as a control. The criteria for inclusion of control participants were healthy patients with normal tissue, no history of betel nut chewing, smoking, or alcohol consumption, did not have any other oral mucosal diseases. Specimens were obtained from the posterior molar pad tissue distal to the third molar. Patient clinical history and general personal information were recorded. The medical ethics committee of the Hainan General Hospital approved this study.

### 2.2 Materials

HUVECs (BNCC Co. Ltd.); fetal bovine serum (10%, HyClone); Penicillin–streptomycin solution (1%, HyClone); Optical microscope (DM2700 M; Leica Microsystems Technology Co. Ltd.); confocal microscopy (Olympus); whole cell lysis reagent (KeyGen Biotech); polyvinylidene fluoride (Beyotime); primary antibodies including anti‐Akt (1:1000, Abcam), anti‐p‐Akt (1:1000, Abcam), anti‐β‐actin (1:1000, CST, Boston), anti‐VEGF (1:1000, Abcam), anti‐Pi3k (1:1000, Abcam), anti‐p‐Pi3k (1:1000, Abcam), anti‐Col‐I (1:1000, CST, Boston); a gel and blot imaging system (Syngene); LY294002 (HY‐10108, MedChemExpress); IGF‐1 (HY‐P700478, MedChemExpress).

## 3 EXPERIMENTAL METHODS

### 3.1 HE dyeing

The buccal OSF and normal tissues were fixed in paraformaldehyde, dehydrated, embedded in paraffin. After solidification, serially sectioned, and hydrated for subsequent pathological observation. Haematoxylin solution was dropped onto the surface of the tissue sections and fixed for 5 min. Subsequently, the staining solution was removed, differentiation solution was added, 1% lithium carbonate solution was used for 1 min and served as a bluing agent, and tissues were stained with eosin for 10 s. The sections were dehydrated, dried and sealed with neutral balsam. The stages and pathological changes in OSF were observed under a microscope.

### 3.2 Masson staining and immunohistochemistry

Tissue sections were stained with Masson's compound solution for 5 min. The slices were treated with phosphomolybdic acid and aniline blue for 5 min, respectively, and stained with differentiation solution for 30 s. Following dehydration, drying, transparency, and sealing, sections were observed under an optical microscope.

Tissue sections were placed in boiling immunohistochemistry (IHC) antigen repair solution (pH 6.0) and cooked for 15 min at low heat. The tissue was then restored at 25°C, and rinsed thrice with PBS. Next, to allow for primary antibody absorption, 3% H_2_O_2_ solution was utilized for 10 min, followed by an incubation at 37°C for 1 h. Subsequently, the tissue sections were incubated with the secondary antibody at 37°C in an incubation box for 30 min. After washed with PBS and stained with DAB solution, the slices were dehydrated, dried, made transparent, and sealed.

### 3.3 qPCR

RNA was extracted TRIzol and purified by RNA purification kit (ThermoFisher Scientific) according to the manufacturer's instructions. Total 1 μg of RNA was utilized to synthesize cDNA with a cDNA synthesis kit (ThermoFisher Scientific). The reaction was performed in a fluorescence quantitative PCR instrument according to the manufacturer's instructions (YEASEN). The primers used are shown in Table 1.

**TABLE 1:** qPCR primers.

Primer	Sequence
Pi3k	F:GAGATTGCAAGCAGTGATAGTG
	R:TAATTTTGGCAGTGATTGTGGG
Akt	F:CAGGAGGAGGAGGAGATGGACTTC
	R:AGGTACTCAAACTCGTTCATGGTCAC
VEGF	F:GGCAATGTGTTGAAGACCTTAG
	R:TCATGCCTCCGAATAAGTACTC
TGF‐β	F:AGCGACTCGCCAGAGTGGTTA
	R:GCAGTGTGTTATCCCTGCTGTCA
GAPDH	F:GGTTGTCTCCTGCGACTTCA
	R:TGGTCCAGGGTTTCTTACTCC

### 3.4 Western blot

After determining the concentration of the protein and unifying the total amount of protein, the samples were lysed for 30 min, and then denatured at 100°C for 10 min. Electrophoresis and membrane translocation were performed for 1 h, the PVDF membranes were soaked in skimmed milk powder. After shaking and blocking on a shaker for 1 h at 25°C, primary antibodies with a diluted concentration of 1:1000 were added. The membrane was incubated at 4°C overnight and incubated with a diluent containing the secondary antibody for 1 h. The PVDF membranes were exposed with a gel and blot imaging system.

### 3.5 Scratching test

Cells were seeded in 6‐well plates. After the cells reached a density of 90%, sterile pipette tips were used to create a gap. After washing with PBS 3 times, the samples were exposed to LY294002 or IGF‐1 with high glucose Dulbecco's modified Eagle's medium (H‐DMEM) containing 1% FBS and 1% (v/v) penicillin–streptomycin. After treatment for 0, 12 and 24 h, scratching healing results were recorded and analysed with Image J.

### 3.6 CCK‐8 assay

Each group was incubated in LY294002 or IGF‐1 for 24 h. The culture medium was substituted with none FBS culture medium containing 10% CCK‐8 liquid and then put in the incubator for more than 2 h. The results were recorded with a absorbance wavelength at 450 nm.

### 3.7 Statistical analysis

All data are represented as mean ± standard deviation (SD). Compare multiple groups in this study were analysed by one‐way analysis of variance (ANOVA), and Spearman's method was used for correlation analysis. The experimental results were collected and analysed using SPSS (version 25.0; SPSS Inc.) and GraphPad Prism software (version 8) and histograms were drawn, *p* < 0.05 was considered statistically significant.

## 4 EXPERIMENTAL RESULTS

### 4.1 Inflammation and collagen level increase as OSF develops

**FIGURE 1:**
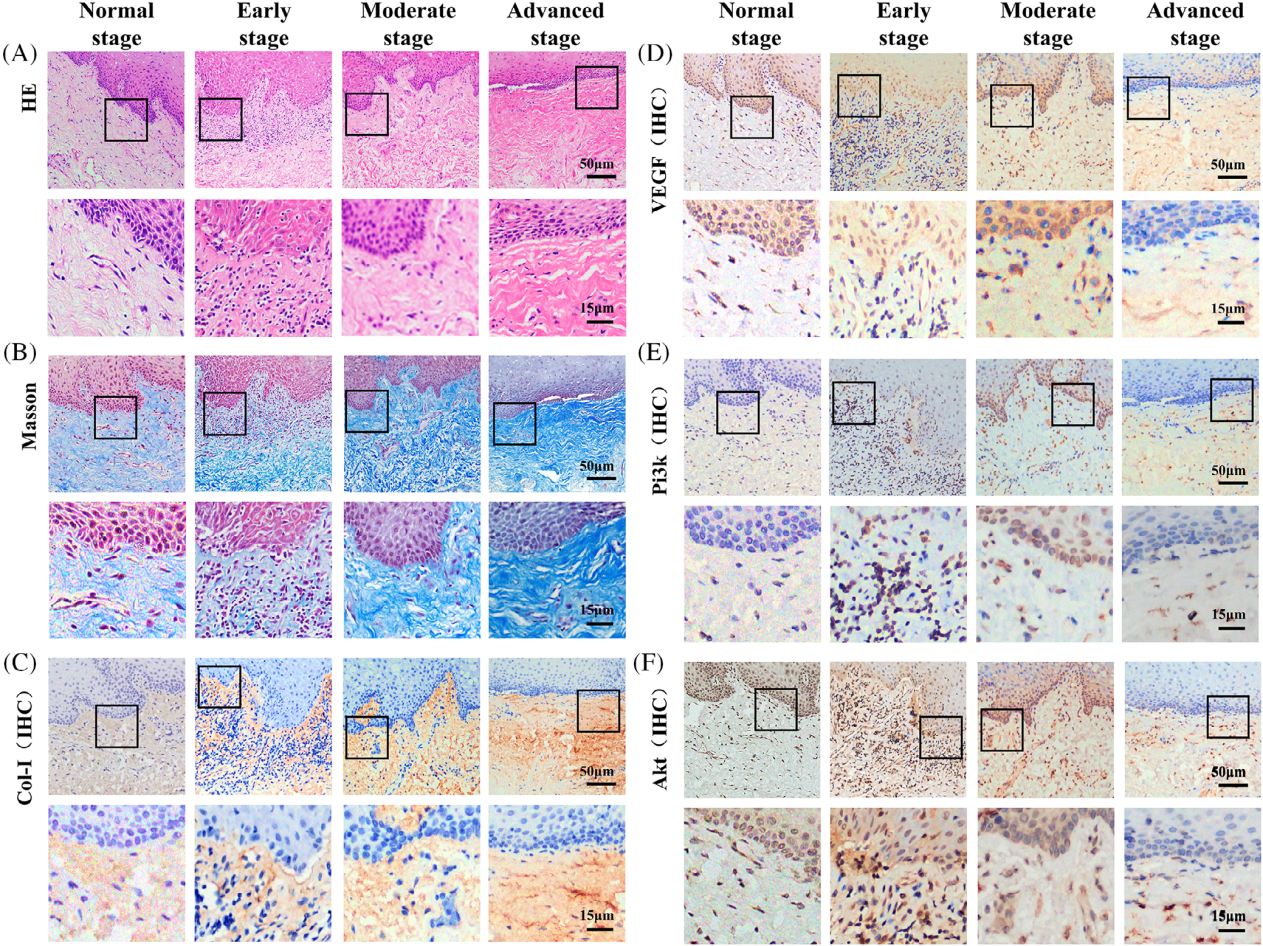
The development of oral submucosal fibrosis and Col‐I, Pi3k, Akt, and VEGF expression during different OSF stages. (A) HE staining and (B) Masson staining of the OSF tissue samples to reveal pathological changes and levels of fibrosis during OSF development. (C) Col‐I expression during OSF progression and a representative field of fibrosis progression. (D) VEGF expression during different stages of OSF. (E, F) Expression of Pi3k and Akt during different OSF stages. (Scale bars = 50 and 15 μm).

The HE staining results (Figure 1A) showed that the early stage OSF tissues had irregular thickening of the epithelial spinous layer with spongy edema, lymphocyte infiltration under the mucosa, collagen edema, capillary proliferation and expansion. In the moderate stage OSF tissues, local epithelial atrophy, submucosal inflammatory cell infiltration, a gradual increase in collagen deposition, normal and contracted blood vessels were observed. In the advanced stage OSF tissues, epithelial atrophy, flat epithelial nail process, infiltration of submucosal inflammatory cells, dense collagen in the lamina propria, and obvious reduction in blood vessels were observed.

**FIGURE 2:**
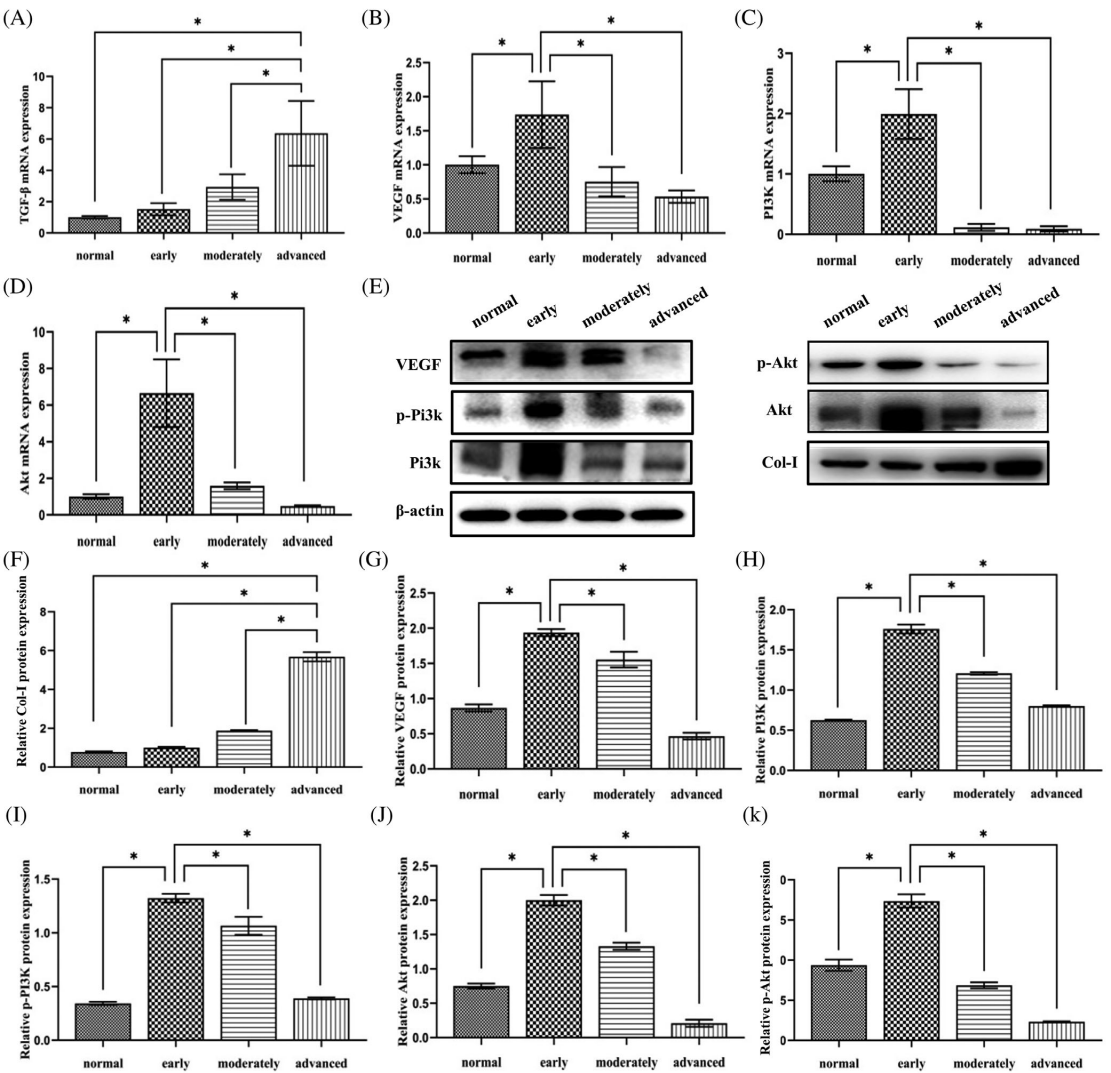
Pi3k/AKT pathway protein and VEGF expression during different OSF stages. (A–D) Histograms of mRNA expression during different OSF stages (E) Western blot images showing Pi3k/Akt pathway, VEGF and Col‐I expression. (F–K) Histograms of mRNA expression during different OSF stages. Data are presented as mean ± standard deviation (SD) (*n* = 3). Statistical analysis: *p < 0.05.

Masson staining of normal to advanced stage OSF tissues showed diffused network of distributed blue‐purple collagen, respectively. These results indicate a gradual increase the level of fibrosis (Figure 2B).

### 4.2 VEGF, Pi3k and Akt expression increase in the early OSF and decrease in middle and late stages

We found that Col‐I expression was closely related to the degree of fibrotic lesions. It was diffusely distributed in the lamina propria of the normal tissues (Figure 1C). In the early‐stage lesions, the density of Col‐I increased, and reticular distribution was observed. In moderate stage tissue lesions, Col‐I was distributed in sheets and gradually increased in scope. In the advanced stage OSF tissues, the lamina propria was covered with brown staining, and the intensity and scope were increased significantly (*p* < 0.05), indicating that Col‐I is distributed in the early, middle, and late stages, and progressively increases with progressing OSF.

VEGF is present in fibroblasts, vascular endothelial cells, and inflammatory cells in the basal layers of the epithelium and submucosa.^9^ In normal tissues, VEGF was mainly expressed in basal cells, and a small amount of VEGF was expressed in vascular endothelial cells in the submucosa (Figure 1D). In the early stage OSF tissues, VEGF expressed more in the basal layer and submucosa than that in normal tissues. With the development of OSF from early stage to advanced stage, VEGF level showed a decreasing trend.

Pi3k and Akt are mainly expressed in inflammatory cells, vascular endothelial cells, and fibroblasts in the basal layer, spinous layer, and epithelial submucosa.^10^, ^11^ Limited Pi3k and Akt were observed in normal tissues (Figure 1E,F). In early stage OSF tissues, high levels of Pi3k and Akt expression are observed in the epithelial basal layer and submucosa. Pi3k and Akt expression decreases as OSF progresses.

### 4.3 Gene expression of TGF‐β increases as OSF develops

TGF‐β mRNA was expressed in normal tissues and in early, moderate, and advanced OSF tissues. The mRNA expression level of TGF‐β in advanced OSF tissues was higher than that observed in healthy tissues as well as in early to moderate OSF tissues. The mRNA expression of TGF‐β was observed between tissue normal and diseased tissue samples (Figure 2A–D). Overall, the mRNA content of VEGF, Pi3k and Akt were more in the early stage OSF tissues. VEGF, Pi3k and Akt decreased as OSF progressed.

### 4.4 The protein level of Col‐I increases as OSF evolves while that of Pi3k, p‐Pi3k, Akt, p‐Akt and VEGF only increase in the early stage

The WB results of Col‐I, Pi3k, p‐Pi3k, Akt, p‐Akt and VEGF showed that these proteins were expressed in all tissues (Figure 2E). Col‐I gradually increased from normal tissue to advanced OSF tissue (Figure 2F). Expression levels of the other 5 proteins were higher in early OSF tissues, compared to that observed in normal tissues and moderate and advanced OSF tissues (Figure 2G–K).

### 4.5 LY294002 inhibited cell migration and IGF‐1 promoted cell migration

**FIGURE 3:**
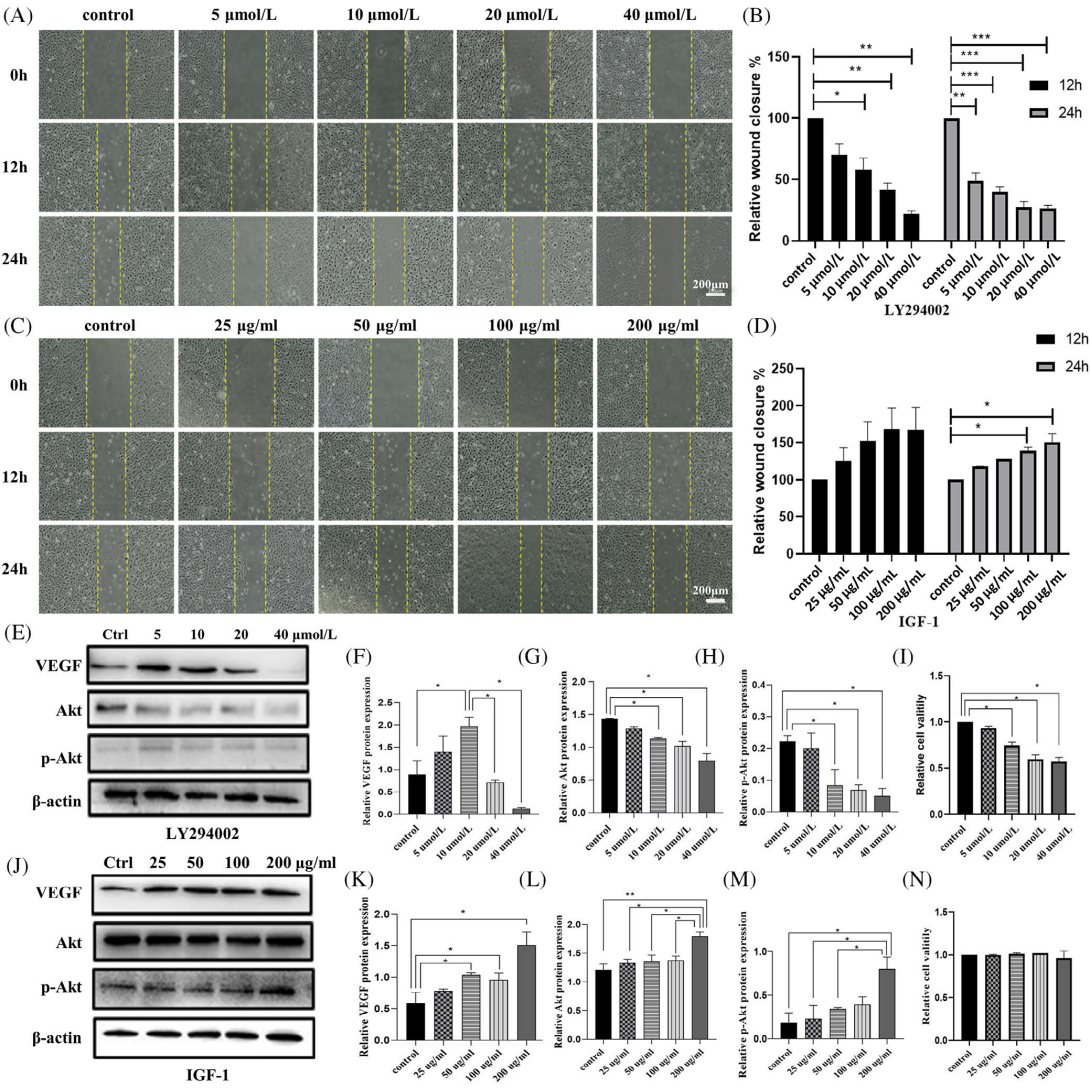
The effects of Pi3k/Akt signalling pathway on cell migration and proliferation. Relative protein expression after LY294002 and IGF‐1 treatment. (A) Images and (B) histograms of scratch tests on HUVECs treated with different concentrations of LY294002 for 0, 12 and 24 h. (C) Images and (D) histograms of scratch tests on HUVECs treated with different concentrations of IGF‐1 for 0, 12 and 24 h. (E‐H) Western Blot images of VEGF, Akt and p‐Akt expression at varying LY294002 concentrations. (J‐M) Western Blot images of VEGF, Akt and p‐Akt expression at varying IGF‐1 concentrations. (I, N) Histograms showing HUVEC viability after treatment with different concentrations of LY294002 and IGF‐1 for 24 h. Data are presented as mean ± standard deviation (SD) (n ≥ 3). Significance: *p < 0.05, **p < 0.01, ***p < 0.001. (Scale bars = 200 μm).

To verify if LY294002 and IGF‐1 would affect cell migration, 12‐ and 24‐h scratch tests were conducted. An optical microscope was used to record human umbilical vein endothelial cells (HUVECs) migration images^12^ (Figure 3A,C). Cell migration was indirectly related to LY294002 concentration. At a concentration of 40 μmol, cell migration was inhibited by 80% (Figure 3B). In contrast, IGF‐1 promoted cell migration. Cells migration was directly proportional to IGF‐1 concentration. Cells migrated 160% at 200 ng/mL (Figure 3D).

### 4.6 LY294002 inhibited cell proliferation

To determine the effect of the drugs on cell proliferation, a CCK8 assay was performed. After 24 h of treatment, LY294002 inhibited proliferation (Figure 3I), whereas IGF‐1 had no significant effect on cell proliferation (Figure 3N).

### 4.7 VEGF expression reached a peak in HUVECs treated with 10 μM LY294002 while Akt and p‐Akt showed a decreasing trend

**FIGURE 4:**
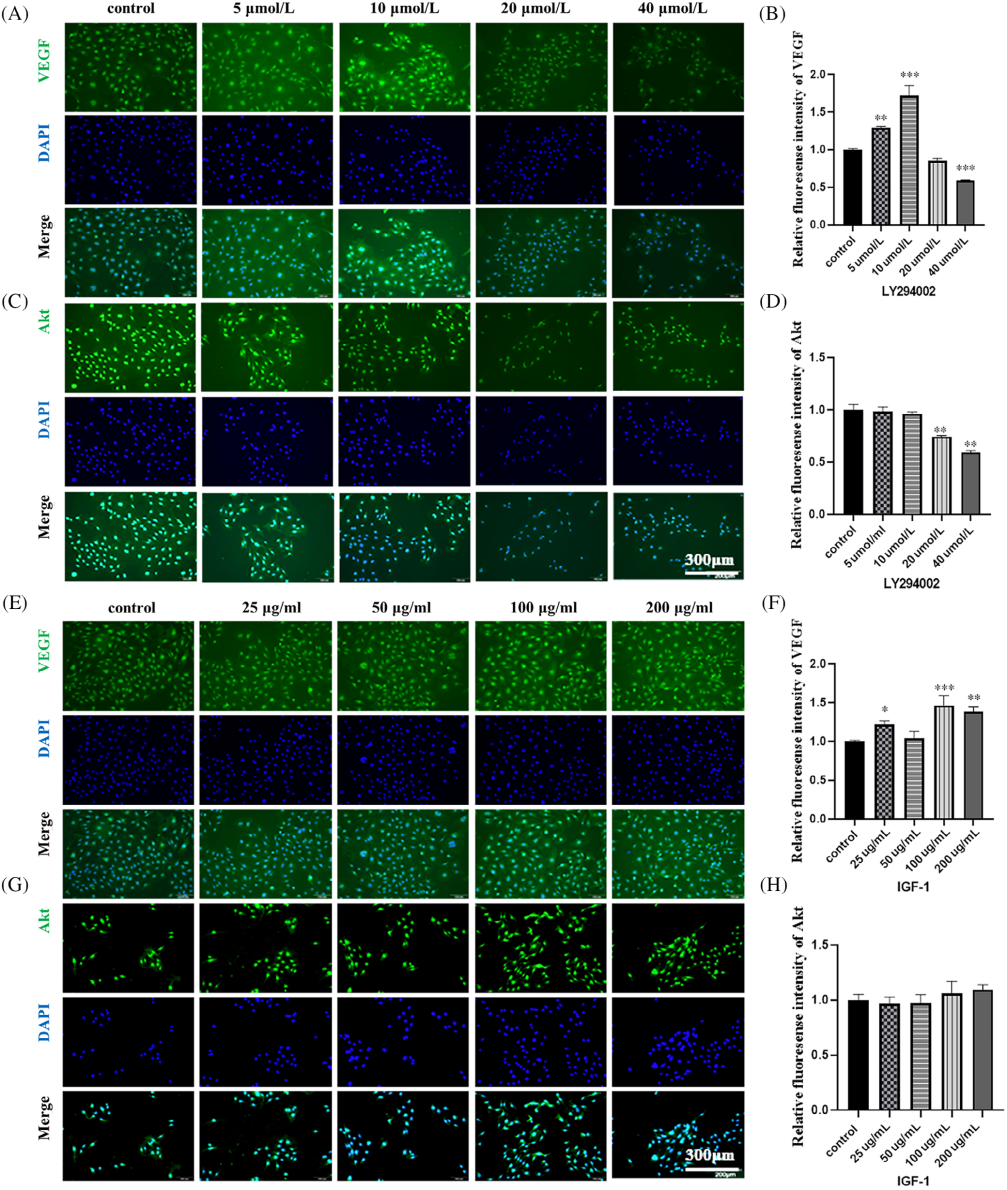
Pi3k/Akt pathway inhibitor (LY294002) and activator (IGF‐1) regulates VEGF and Akt expression at different concentrations. (A, B) VEGF fluorescence and expression after LY294002 treatment at varying doses. (C, D) Akt fluorescence and expression after LY294002 treatment at varying doses. (E, F) VEGF fluorescence and expression after IGF‐1 treatment at varying doses. (G, H) Akt fluorescence and expression after IGF‐1 treatment at varying doses.

In LY294002 group, VEGF expression increased in the concentration of 10 μM compared to that from control group. Thereafter, VEGF expression significantly decreased with an increase in LY294002 concentration (Figure 3E,F), which is accordance to the IF results. The expression level of Akt and p‐Akt in the 10, 20 and 40 μM groups was lower compared to the control group. With an increase in LY294002 concentration, Akt and p‐Akt proteins showed a decreasing trend (Figure 3G,H), which is also found in immunofluorescence experiments of HUVECs (Figure 4A–D).

### 4.8 VEGF, Akt and p‐Akt showed an increase with IGF‐1 increased

In IGF‐1 group, VEGF expression from WB results increased before the concentration of IGF‐1 reached at 50 μg/mL. Although there is a fluctuation at 100 μg/mL, VEGF expression still reached a peak 100 μg/mL (Figure 3J,K). Akt and p‐Akt expression was the highest in the 200 ng/mL group (Figure 3L,M). The IF results showed that VEGF expression reached highest 1.5‐fold of control group at 100 μg/mL, although some fluctuation (Figure 4E,F). And Akt level increased slightly with the increased concentration of IGF‐1, reaching 1.1‐fold at 200 μg/mL (Figure 4G,H).

## 5 DISCUSSION

In lamina propria and submucosa, stenosis and decreased blood vessels are one of the main pathological features of OSF. This pathological change in vascular occlusion and reduction eventually leads to the continuous accumulation of locally formed collagen, which cannot be ameliorated through blood circulation. Moreover, systemic nutrients and therapeutic drugs cannot reach the lesion area, resulting in the poor OSF treatment efficacy. A new therapeutic approach for OSF should focus on vascular endothelial cell protection, which would avoid the repair of uncontrolled fibrosis. Recently, a novel nano material known as tetrahedral framework nucleic acid has been proved efficiently on treating fibrosis related diseases.^13^, ^14^, ^15^

VEGF is the core factor of endothelial cells and angiogenesis, and its main role is to mediate inflammation, proliferation and migration, promote angiogenesis, and participate in the degradation of the extracellular matrix.^16^, ^17^ In this study, we studied the role of VEGF in the pathogenesis of OSF. VEGF was expressed in normal tissues, and higher expression was observed in the early stage OSF tissues. Lower VEGF expression was observed in the moderate and advanced stage OSF tissues. In early, moderate, and advanced stage OSF tissues, capillary proliferation and dilation; vasoconstriction; and epithelial atrophy, flattened epithelial pegs process, and significantly reduced blood vessels were observed, respectively. These results indicate that in response to local tissue ischemia, VEGF is secreted by basal cells and induces endothelial cell migration to the cortex to maintain epithelium blood supply, which is similar to that shown in a previous study.^18^ The increased VEGF expression in early stage OSF may be caused by tissue hypoxia following epithelial vascular injury, which activates HIF‐I, upregulates VEGF expression, and triggers angiogenesis. Some inflammatory cytokines such as IL‐1, IL‐6 and TNF‐α can also induce VEGF expression.^19^ In addition, increased VEGF expression may be a tissue compensatory response to improve tissue ischemia and hypoxia.^20^ We found that with OSF progression, VEGF expression decreases due to collagen aggravation and the occlusion of blood vessels. Arecoline causes basal cell damage and induces dysplasia, and a decrease in cell number reduces VEGF secretion.^21^ Cell number constantly reduces in the submucosa, and the secretion of vascular growth suppressor factors also decrease VEGF expression. Husain et al. found that, in renal fibrosis in mice, fewer endothelial cells and decreased VEGF expression aggravates renal tissue fibrosis, while increased VEGF expression induces angiogenesis, reduces the inflammatory response, and prevents fibrosis progression.^22^ Therefore, decreased VEGF expression in OSF leading to ischemia, may be key to OSF progression.

Pi3k is a class of lipid kinases responsible for coordinating the performance of various cells, and affects cell proliferation, differentiation, apoptosis, and angiogenesis.^23^, ^24^, ^25^ AKT1 is a downstream molecule of the Pi3k pathway and exists in a low‐activity state in the cytoplasm. Activated p‐Akt is the transduction centre of multiple pathways and is of vital significance in cell proliferation and angiogenesis.^26^ The key proteins in the Pi3k/Akt signalling pathway were found to be upregulated in the early OSF stage but downregulated in the moderate and advanced stages. Protein upregulation in early OSF may be caused by mucosal tissue injury, resulting in a stress response, and cytokine over secretion, which activates the Pi3k/Akt pathway, promotes vascular endothelial cell proliferation and migration, inhibits epithelial and endothelial cell apoptosis, and prevents further tissue damage. The expression of Pi3k, Akt, and their phosphorylated forms decreased in moderate and advanced OSF stages, which may be due to collagen accumulation and hyaline alteration resulting in a decrease in epithelial and endothelial cells, leading to a decrease in the secretion of Pi3k and Akt. At different stages of OSF, the protein expression of Pi3k/Akt pathway‐related signalling changed, suggesting their involvement in the occurrence and progress of OSF.

Recently, researchers have found that the Pi3k/Akt signalling pathway regulates the main functions of cell metabolism, proliferation, survival, and other functions by regulating the VEGF gene.^27^ It has shown the function of regulating VEGF expression in myocardial ischemia reperfusion injury, myocardial infarction, and participates in the generation of blood vessels.^28^, ^29^, ^30^ In vitro experiments have revealed that VEGF protein expression, in HUVECs, is modulated by Pi3k/Akt pathway regulation. Considering all the results, we believe that Pi3k/Akt is involved in OSF development, and confirm the presence of an interplay between Pi3k/Akt and VEGF, which indicates a potential OSF therapeutic target. VEGF, Pi3k, and Akt expression positively correlated to OSF disease state, indicating that the Pi3k/Akt pathway and VEGF are involved in the pathogenesis of OSF lesions, and work synergistically in the development of fibrosis. In this study, LY294002, a Pi3k/Akt pathway inhibitor, regulated VEGF secretion, VEGF expression positively and negatively correlated with LY294002 below and above a concentration 10 μM, respectively. IGF‐1, a Pi3k/Akt pathway activator,^31^ was positively correlated with VEGF expression, indicating that Pi3k/Akt pathway regulates VEGF secretion.^32^ The observed vessel structure in OSF lesions was consistent with VEGF, Pi3k, and Akt expression, further suggesting previous points. Zhou et al. found that, in a rat model of myocardial ischemia–reperfusion injury, p‐Akt and VEGF expression was upregulated, and further increased after high mobility group box 1 (HMGB1) treatment. They also found that the Pi3k/Akt pathway could upregulate VEGF expression, promote blood vessel formation, improve cardiac function, and thus protect the heart.^33^ Targeted activation of the Pi3k/Akt pathway and increase of VEGF expression can protect tissues and organs.

Most oral tissues of patients with OSF are affected by fibrosis, resulting in the accumulation of subepithelial and submucosal fibres in the oral mucosa and deep tissues, which restricts mouth opening and tongue movement, dysphagia, and speech impediments. Therefore, the goal of treatment is to alleviate these symptoms and prevent or reverse further fibrotic changes.^13^ However, owing to the clinical manifestations of OSF, treatment to restore the function and aesthetics remains a challenge. Currently, vasodilators are the most used and feasible OSF treatment option, however, the systemic drugs offer non‐specific targeting and are associated with numerous side‐effects.^34^ The forced activation of Pi3k/Akt pathway via active protein mutants, in endothelial cells leads to neovascularization, and a blockade of this pathway leads to the vascular degradation in multiple organs. Therefore, angiogenesis promotion through long‐term VEGF regulation via Pi3k/Akt pathway regulation is a promising therapeutic strategy.^35^ Li et al have reported that a novel bioswitchable miRNA inhibitor delivery system (BiRDS) has the capability and editability to improve the stability and delivery efficacy of miRs, which means BiRDS may be able to carry miRNA targeting fibrosis into skin tissue, providing a promising treatment for OSF.^36^ Unfortunately, the specific mechanism in OSF remains unclear and requires further investigation. Nevertheless, due to the synergistic effect between Pi3k/Akt pathway and VEGF in OSF lesions and fibrosis,^37^ targeted Pi3k/Akt pathway regulation can induce VEGF expression and improve ischemia, ultimately treating OSF and associated symptoms.

(A) Images and (B) histograms of scratch tests on HUVECs treated with different concentrations of LY294002 for 0, 12 and 24 h. (C) Images and (D) histograms of scratch tests on HUVECs treated with different concentrations of IGF‐1 for 0, 12 and 24 h. (E–H) Western Blot images of VEGF, Akt and p‐Akt expression at varying LY294002 concentrations. (J‐M) Western Blot images of VEGF, Akt and p‐Akt expression at varying IGF‐1 concentrations. (I, N) Histograms showing HUVEC viability after treatment with different concentrations of LY294002 and IGF‐1 for 24 h. Data are presented as mean ± standard deviation (SD) (*n* ≥ 3). Significance: *p < 0.05, **p < 0.01, ***p < 0.001. (Scale bars = 200 μm).

## AUTHOR CONTRIBUTIONS

All authors have made important contributions to this study. Yanan Lin conducted ex vitro experiments, sorted data, and wrote the main manuscript. Yueying Jiang collected and analysed the data, designed the schematic diagram, and wrote the manuscript. Haiyu Xian conducted in vitro experiments and analysed the data. Tao Wang and Xiaoxiao Cai conceived and initiated the study, analysed the data, provided funding and proofread the manuscript. All authors have read and approved the manuscript.

## FUNDING INFORMATION

This study was supported by National Natural Science Foundation of China (No. 81960199) and Hainan Province Science and Technology special Fund (No. ZDYF2021SHFZ114).

## CONFLICT OF INTEREST STATEMENT

The authors declare that there is no conflict of interest.

## PATIENT CONSENT STATEMENT

Investigators have obtained informed consent before enrolling participants in the study.

## Data Availability

Data will be made available on request.

## References

[1] Cai X , Yao Z , Liu G , Cui L , Li H , Huang J . Oral submucous fibrosis: a clinicopathological study of 674 cases in China. J Oral Pathol Med. 2019;48:321‐325. doi:10.1111/jop.12836 30715767 PMC6593413

[2] Shih YH , Wang TH , Shieh TM , Tseng YH . Oral submucous fibrosis: a review on etiopathogenesis, diagnosis, and therapy. Int J Mol Sci. 2019;20:2940. doi:10.3390/ijms20122940 31208114 PMC6627879

[3] Shen YW , Shih YH , Fuh LJ , Shieh TM . Oral submucous fibrosis: a review on biomarkers, pathogenic mechanisms, and treatments. Int J Mol Sci. 2020;21:7231. doi:10.3390/ijms21197231 33008091 PMC7582467

[4] Wigley FM . Vascular disease in scleroderma. Clin Rev Allergy Immunol. 2009;36:150‐175. doi:10.1007/s12016-008-8106-x 19067252

[5] Coultas L , Chawengsaksophak K , Rossant J . Endothelial cells and VEGF in vascular development. Nature. 2005;438:937‐945. doi:10.1038/nature04479 16355211

[6] Wang J , Wu M . The up‐regulation of miR‐21 by gastrodin to promote the angiogenesis ability of human umbilical vein endothelial cells by activating the signaling pathway of Pi3k/Akt. Bioengineered. 2021;12:5402‐5410. doi:10.1080/21655979.2021.1964895 34424813 PMC8806924

[7] He H , Zhang H , Pan Y , et al. Low oxygen concentration improves yak oocyte maturation and inhibits apoptosis through HIF‐1 and VEGF. Reprod Domest Anim. 2022;57:381‐392. doi:10.1111/rda.14076 34967955

[8] Karar J , Maity A . Pi3k/AKT/mTOR pathway in angiogenesis. Front Mol Neurosci. 2011;4:51. doi:10.3389/fnmol.2011.00051 22144946 PMC3228996

[9] Shams F , Moravvej H , Hosseinzadeh S , et al. Overexpression of VEGF in dermal fibroblast cells accelerates the angiogenesis and wound healing function: in vitro and in vivo studies. Sci Rep. 2022;12:18529. doi:10.1038/s41598-022-23304-8 36323953 PMC9630276

[10] Guo Z , Wang Y , Wen X , Xu X , Yan L . Beta‐klotho promotes the development of intrauterine adhesions via the Pi3k/AKT signaling pathway. Int J Mol Sci. 2022;23:11294. doi:10.3390/ijms231911294 36232594 PMC9569898

[11] Danussi C , Petrucco A , Wassermann B , et al. EMILIN1‐alpha4/alpha9 integrin interaction inhibits dermal fibroblast and keratinocyte proliferation. J Cell Biol. 2011;195:131‐145. doi:10.1083/jcb.201008013 21949412 PMC3187715

[12] Kreuger J , Phillipson M . Targeting vascular and leukocyte communication in angiogenesis, inflammation and fibrosis. Nat Rev Drug Discov. 2016;15(2):125‐142. doi:10.1038/nrd.2015.2 26612664

[13] Jiang Y , Li S , Zhang T , et al. Tetrahedral framework nucleic acids inhibit skin fibrosis via the pyroptosis pathway. ACS Appl Mater Interfaces. 2022;14:15069‐15079. doi:10.1021/acsami.2c02877 35319864

[14] Tian T , Zhang T , Shi S , Gao Y , Cai X , Lin Y . A dynamic DNA tetrahedron framework for active targeting. Nat Protoc. 2023;18(4):1028‐1055. doi:10.1038/s41596-022-00791-7 36670289

[15] Tian T , Li Y , Lin Y . Prospects and challenges of dynamic DNA nanostructures in biomedical applications. Bone Res. 2022;10(1):40. doi:10.1038/s41413-022-00212-1 35606345 PMC9125017

[16] Zhang J , Chu M . Differential roles of VEGF: relevance to tissue fibrosis. J Cell Biochem. 2019;120:10945‐10951. doi:10.1002/jcb.28489 30793361

[17] Pulkkinen HH , Kiema M , Lappalainen JP , et al. BMP6/TAZ‐Hippo signaling modulates angiogenesis and endothelial cell response to VEGF. Angiogenesis. 2021;24:129‐144. doi:10.1007/s10456-020-09748-4 33021694 PMC7921060

[18] Saxena A , Walters MS , Shieh JH , et al. Extracellular vesicles from human airway basal cells respond to cigarette smoke extract and affect vascular endothelial cells. Sci Rep. 2021;11:6104. doi:10.1038/s41598-021-85534-6 33731767 PMC7969738

[19] Adekoya TO , Richardson RM . Cytokines and chemokines as mediators of prostate cancer metastasis. Int J Mol Sci. 2020;21:4449. doi:10.3390/ijms21124449 32585812 PMC7352203

[20] Choudhari SS , Kulkarni DG , Patankar S , et al. Angiogenesis and fibrogenesis in oral submucous fibrosis: a viewpoint. J Contemp Dent Pract. 2018;19:242‐245. doi:10.5005/jp-journals-10024-2244 29422478

[21] Sharma M , Shetty SS , Radhakrishnan R . Oral submucous fibrosis as an overhealing wound: implications in malignant transformation. Recent Pat Anticancer Drug Discov. 2018;13:272‐291. doi:10.2174/1574892813666180227103147 29485009

[22] Husain SA , King KL , Batal I , et al. Reproducibility of deceased donor kidney procurement biopsies. Clin J Am Soc Nephrol. 2020;15:257‐264. doi:10.2215/CJN.09170819 31974289 PMC7015101

[23] Xie Y , Shi X , Sheng K , et al. Pi3k/Akt signaling transduction pathway, erythropoiesis and glycolysis in hypoxia (review). Mol Med Rep. 2019;19:783‐791. doi:10.3892/mmr.2018.9713 30535469 PMC6323245

[24] Tewari D , Patni P , Bishayee A , Sah AN , Bishayee A . Natural products targeting the Pi3k‐Akt‐mTOR signaling pathway in cancer: a novel therapeutic strategy. Semin Cancer Biol. 2022;80:1‐17. doi:10.1016/j.semcancer.2019.12.008 31866476

[25] Noorolyai S , Shajari N , Baghbani E , Sadreddini S , Baradaran B . The relation between Pi3k/AKT signalling pathway and cancer. Gene. 2019;698:120‐128. doi:10.1016/j.gene.2019.02.076 30849534

[26] Cui LH , Li CX , Zhuo YZ , Yang L , Cui NQ , Zhang SK . Saikosaponin d ameliorates pancreatic fibrosis by inhibiting autophagy of pancreatic stellate cells via Pi3k/Akt/mTOR pathway. Chem Biol Interact. 2019;300:18‐26. doi:10.1016/j.cbi.2019.01.005 30611790

[27] Sun Y , Wang T , Wen QT , Yu DH , Chen JX . VEGF gene transfection restores the angiogenesis of oral submucous fibrosis in mice. Ann Transl Med. 2021;9:930. doi:10.21037/atm-21-2213 34350245 PMC8263869

[28] Fu J , Tang Y , Zhang Z , Tong L , Yue R , Cai L . Gastrin exerts a protective effect against myocardial infarction via promoting angiogenesis. Mol Med. 2021;27:90. doi:10.1186/s10020-021-00352-w 34412590 PMC8375043

[29] Cheng S , Zhang X , Feng Q , et al. Astragaloside IV exerts angiogenesis and cardioprotection after myocardial infarction via regulating PTEN/Pi3k/Akt signaling pathway. Life Sci. 2019;227:82‐93. doi:10.1016/j.lfs.2019.04.040 31004658

[30] Zhou YH , Han QF , Gao L , et al. HMGB1 protects the heart against ischemia‐reperfusion injury via Pi3k/AkT pathway‐mediated upregulation of VEGF expression. Front Physiol. 2019;10:1595. doi:10.3389/fphys.2019.01595 32063860 PMC7000523

[31] Yoshida T , Delafontaine P . Mechanisms of IGF‐1‐mediated regulation of skeletal muscle hypertrophy and atrophy. Cells. 2020;9(9):1970. Published Aug 26, 2020. doi:10.3390/cells9091970 32858949 PMC7564605

[32] Li F , Liu Y , Ren L , Sun Q , Luo YX . IGF‐1 regulates ang II and VEGF signaling pathways in retinal neovascularization. Eur Rev Med Pharmacol Sci. 2018;22:6175‐6180. doi:10.26355/eurrev_201810_16022 30338783

[33] Zhou Y , Li Y , Mu T . HMGB1 neutralizing antibody attenuates cardiac injury and apoptosis induced by hemorrhagic shock/resuscitation in rats. Biol Pharm Bull. 2015;38:1150‐1160. doi:10.1248/bpb.b15-00026 26040987

[34] Zhang Y , Wang X , Liu R , et al. The effectiveness and safety of nicorandil in the treatment of patients with microvascular angina: a protocol for systematic review and meta‐analysis. Medicine. 2021;100:e23888. doi:10.1097/MD.0000000000023888 33466132 PMC7808505

[35] Chen R , Wen D , Fu W , et al. Treatment effect of DNA framework nucleic acids on diffuse microvascular endothelial cell injury after subarachnoid hemorrhage. Cell Prolif. 2022;55(4):e13206. doi:10.1111/cpr.13206 35187748 PMC9055902

[36] Li S , Liu Y , Zhang T , et al. A tetrahedral framework DNA‐based bioswitchable miRNA inhibitor delivery system: application to skin anti‐aging. Adv Mater. 2022;34(46):e2204287. doi:10.1002/adma.202204287 35901292

[37] Zhao X , Ren Y , Ren H , et al. The mechanism of myocardial fibrosis is ameliorated by myocardial infarction‐associated transcript through the Pi3k/Akt signaling pathway to relieve heart failure. J Int Med Res. 2021;49:3000605211031433. doi:10.1177/03000605211031433 34275376 PMC8293849

